# Current e-cigarette use among in-school adolescents in West Malaysia: Examining the interactions between sociodemographic characteristics and lifestyle risk behaviours

**DOI:** 10.1371/journal.pone.0263355

**Published:** 2022-01-31

**Authors:** Miaw Yn Jane Ling, Norfazilah Ahmad, Muhammad Fadhli Mohd Yusoff, Kuang Hock Lim

**Affiliations:** 1 Department of Community Health, Faculty of Medicine, Universiti Kebangsaan Malaysia, Kuala Lumpur, Malaysia; 2 Institute for Public Health, National Institutes of Health, Ministry of Health Malaysia, Selangor, Malaysia; 3 Institute for Medical Research, National Institutes of Health, Ministry of Health Malaysia, Kuala Lumpur, Malaysia; UCSI University, MALAYSIA

## Abstract

**Background:**

Adolescent e-cigarette use has increased dramatically. Most e-cigarette liquids contain nicotine, which can harm the developing adolescent brain. Local studies examining the risk factors of adolescent e-cigarette use and interactions between its risk factors are limited. This study was aimed at determining the prevalence of current e-cigarette use and its associated factors among in-school adolescents in West Malaysia. We also examined the possible sociodemographic characteristic, lifestyle risk behaviour and parental factor interactions that affect the probability of current e-cigarette use.

**Methods:**

We conducted a cross-sectional study using data from the National Health and Morbidity Survey 2017. Respondents aged 13–18 years were included in the study. The data were analysed using STATA (v.15).

**Results:**

The prevalence of current e-cigarette use was 9.1%. Male gender, older age, Malay ethnicity, schooling in urban area, current smoking, current alcohol use, current drug use, having parents that are not married and living together and parental tobacco product use were significantly associated with current e-cigarette use. There were significant interactions between: i) gender with age, ethnicity, current smoking, current alcohol use and current drug use, ii) ethnicity with current smoking and current alcohol use, iii) locality with ethnicity, current smoking and current alcohol use, iv) current drug use with age, ethnicity, current smoking and current alcohol use, v) parental marital status with gender, age and ethnicity, and vi) parental tobacco use with ethnicity and current smoking.

**Conclusion:**

Our findings identify significant associations between sociodemographic characteristics, lifestyle risk behaviours and parental factors with current e-cigarette use. They also provide new insight into the interactions between these factors that affect the probability of current e-cigarette use among West Malaysian adolescents. Efforts to tackle e-cigarette use in Malaysian adolescents should target sociodemographic characteristics, lifestyle risk behaviours and parental factors such as smoking cessation intervention for parents.

## Introduction

According to the World Health Organization (WHO), adolescents are individuals aged 10–19 years [1]. Adolescents are susceptible to risk-taking behaviours such as risky sexual behaviour, alcohol abuse and substance use problems [2]. Adolescent e-cigarette use has increased dramatically. E-cigarettes are battery-powered devices that allow the users to inhale nicotine through a vapour. These products use a liquid solution that often contains nicotine and that comprises propylene glycol, glycerine, flavouring agents and additives [3]. Between 2011 and 2015, there was an alarming 900% increase in e-cigarette use among adolescents in the United States [3]. Most e-cigarette liquids, including those labelled nicotine-free, actually contain nicotine [4], which is highly addictive and harmful to the developing adolescent brain [3]. Most adolescents use e-cigarettes because they want to try something new, like the taste and smell, and feel that e-cigarettes are popular and safer than conventional cigarettes, while others use e-cigarettes as a way to quit smoking [5, 6], despite the fact that the existing evidence of e-cigarettes’ effectiveness as a quit smoking tool remains equivocal [7–9].

There have been studies aimed at understanding the factors that influence adolescent e-cigarette use. Individual risk factors such as being male [10], older [11] and an urban dweller [12] were associated with e-cigarette use among adolescents. The evidence also shows that e-cigarette use is associated with cigarette smoking [13], alcohol use and drug use [12], indicating that e-cigarette use may be a gateway to using other substances in adolescents. Another study found that adolescents with friends who smoked were more likely to be e-cigarette users [12]. Apart from that, the literature has highlighted that parental tobacco product use [5, 14], poor parental monitoring [15] and parental divorce [16] were associated with e-cigarette use, while having good parental connectedness [17] was protective against e-cigarette use among adolescents.

With growing concern over adolescent e-cigarette use, more research is needed to better understand the associations between e-cigarette use with certain risk factors and whether certain groups of adolescents may be more likely to engage in concurrent use of substances. A recent study highlighted the moderating effect of age and sex in the association between e-cigarette use and other risk factors. For example, female e-cigarette users were more likely to use alcohol and drugs compared to males, while younger e-cigarette users were more likely to smoke and use drugs compared with older adolescents [18]. Further, a study on e-cigarette use among high school students in the United States reported a significant interaction between locality and cigarette smoking; urban cigarette smokers were more likely to use e-cigarettes than rural cigarette smokers [19]. Additionally, it was recently reported that ethnicity acts as an effect modifier in the link between the use of e-cigarettes at school level and student level [20].

Studies on e-cigarette use in Malaysia have focused on the adult population [21, 22], while studies among adolescents are limited. In Malaysia, the prevalence of current e-cigarette use among adolescents increased from 1.2% in 2012 [23] to 9.1% in 2016 [24]. A local study reported that being male, older, Malay, and a cigarette smoker were associated with adolescent e-cigarette use [6]. Apart from that, a study involving university students in Malaysia also reported a significant association between gender and e-cigarette use [25]. Nevertheless, local studies exploring the association between lifestyle risk behaviours (cigarette smoking, alcohol drinking, drug use) and parental factors (parental marital status, parental monitoring, parental tobacco product use) with e-cigarette use among adolescents are lacking. Furthermore, local studies examining the interactions between the factors of e-cigarette use among adolescents are also limited. Obtaining this information locally is important due to the differences in legislation, smoking norms, culture and parenting styles. Better identification of adolescents at risk of e-cigarette use is also essential for supporting the development and implementation of interventions for addressing e-cigarette use among Malaysian adolescents. Considering the complex nature of e-cigarette use in adolescents, the present study was aimed at determining: (1) the prevalence of current e-cigarette use and its associated factors among in-school adolescents in West Malaysia, and (2) the possible sociodemographic characteristic, lifestyle risk behaviour and parental factor interactions that could affect the probability of current e-cigarette use among in-school adolescents in West Malaysia.

## Materials and methods

### Study setting

West Malaysia consists of 11 states (Perlis, Kedah, Penang, Perak, Kelantan, Terengganu, Pahang, Selangor, Negeri Sembilan, Malacca, Johor) and two federal territories (Kuala Lumpur, Putrajaya), with an estimated population of 25.9 million in 2020 [26]. This study included all 11 states and the two federal territories in West Malaysia.

### Study design and population

The NHMS 2017 was a cross-sectional study which used two-stage stratified cluster sampling design to select a representative sample of secondary in-school adolescents in Malaysia [27]. The sample size was calculated for all study objectives and the respondents were sampled based on the highest sample size calculated. The sample size was calculated for the analysis at the state level (strata level). The total sample size for the study was the summation of sample from all the states.

West Malaysia was stratified into 11 states and two federal territories. The first stage of sampling was the random selection of secondary schools with probability proportionate to school enrolment size. Probability proportion to size is a sampling procedure under which the probability of a unit being sampled is proportional to the size of the ultimate unit, giving larger clusters a greater probability of selection and smaller clusters a lower probability of selection. Based on the required sample size for each stratum, the following steps were applied for each stratum: (i) A list of primary sampling units (schools) and their population size was developed; (ii) The cumulative sum of the population size was determined; (iii) The number of clusters (*d*) (schools) to be sampled was determined; (iv) The number of respondents to be sampled from each cluster was determined (the same number of respondents was sampled from each cluster to ensure that all individuals have the same probability of selection regardless of the size of their cluster); (v) Obtain the sampling interval (*SI*) by dividing the total population by the number of clusters to be sampled; (vi) A random number between 1 and the *SI* was chosen (this is the random start (*RS*)); (vii) The following series were calculated: *RS*; *RS* + *SI*; *RS* + 2*SI*; …. *RS* + (*d*-1)*SI; (viii) The probability for each cluster being sampled: Prob 1 = (*a* x *d*) / *b* (*a* = cluster population; *b* = total population; *d* = number of clusters). Prob 1 is proportionate to the value of “*a*” (number of students in the clusters).

The second stage of sampling was the selection of classes in the selected schools by using the systematic random sampling. As mentioned earlier, in order to ensure that all individuals in the population have the same probability of selection irrespective of the size of their cluster, the same number of individuals were sampled from each cluster. The following steps were done for each selected schools: (i) The average number of students in a class from form 1 to 5 was determined and the number of classes to be sampled was determined accordingly to obtain the required number of students from each school; (ii) The sampling interval (*SI*) was determined by dividing the total number of classes with the number of classes to be sampled; (iii) A random number for the start of selection (*RS*) was determined; (iv) Classes were selected accordingly based on the SI. A total of 176 secondary schools were selected from West Malaysia and subsequently four to 10 classes were selected from each selected school to participate in the NHMS 2017. The present study was a cross-sectional study involving in-school adolescents in West Malaysia (including 11 states and 2 federal territories). Respondents aged 13–18 years were included in the present study.

### Study instrument

The NHMS 2017 used a self-administered bilingual questionnaire adapted from the Malaysian Global School-based Student Health Survey (GSHS) 2012 [23]. The questionnaire was finalized by a panel of experts who are familiar with the areas covered under the Malaysian GSHS 2012. The variables included in this study were extracted from the NHMS 2017 database with a set of data sheets.

### Outcome variable

The dependent variable in our study (current e-cigarette use) was defined as the use of e-cigarettes in the past 30 days [27].

### Independent variables

The independent variables were gender (male, female), age (years; 13–15, 16–18), locality (urban, rural), ethnicity (Malay, non-Malay), current smoking (yes, no), current alcohol use (yes, no), current drug use (yes, no), parents’ marital status (married and living together, other), parent(s) use of any tobacco product (one or both parents, none) and parental supervision in the past 30 days (yes, no).

Locality was categorized based on the school location [27], while the age cut-off was chosen based on the age of lower and upper secondary school students [6]. The ethnic groups (Malay, non-Malay) have been used in another local study involving Malaysian adolescents [28]. Respondents who were Chinese, Indian or Indigenous peoples of Malaysia were classified as non-Malay.

Current smoking was defined as the use of any smoked tobacco products in the past 30 days, including manufactured cigarettes, roll-your-own cigarettes, traditional hand-rolled cigarettes, shisha, cigar or pipe [27]. Current alcohol use was defined as having at least a ‘drink’ of alcohol (a glass of wine, tuak, lihing, bahar, ijuk or toddy; a can of beer, a small glass of liquor or mixed drink) in the past 30 days [27]. Those who used drugs in the past 30 days, including heroin, morphine, glue, amphetamine/methamphetamines and marijuana, were categorized as current drug users [27].

Parents’ marital status was categorized into ‘married and living together’ and ‘other’ (i.e. married and living apart, divorced, separated, widow, widower) [29], while parent(s) use of any tobacco product was defined as the use of any tobacco product, including manufactured cigarettes, roll-your-own cigarettes, traditional hand-rolled cigarettes, shisha, cigar, pipe, e-cigarettes, chewing tobacco or snuff [27]. Adolescents with parents who had always or most of the time checked to see if their homework was done in the past 30 days were categorized as having parental supervision in the past 30 days [27].

### Statistical analysis

The data were analysed using STATA (v.15). Descriptive statistics of frequencies (*n*) and percentages (%) were used for qualitative data, while the appropriate measure of central tendency was used for quantitative data. The prevalence of current e-cigarette use was estimated using the percentage (%) and its corresponding 95% confidence interval (CI). Chi-square analysis was performed to determine the association between current e-cigarette use with all the independent variables. The associations between current e-cigarette use with all the independent variables were tested with simple logistic regression to obtain the crude odds ratio (OR). Variables with *p* < 0.25 [30] were included in multiple logistic regression analysis to obtain the final model and the adjusted OR (AOR) after controlling for potential confounders. The final model was tested for all possible two-way interactions (multiplicative interaction) between the independent variables, and its fitness was assessed. From the final model fitted with significant interaction terms, the predicted probabilities for current e-cigarette use were computed using the margin command to obtain the average predictive probability values. Graphs were plotted using the marginsplot command [31, 32].

### Ethical approval

The data for this study were obtained from the Malaysian National Health and Morbidity Survey (NHMS) 2017 [27], a cross-sectional, national school-based study that assesses the prevalence of health risk behaviours and protective factors among secondary in-school adolescents in Malaysia. The NHMS 2017 had obtained approval from the Ministry of Health Malaysia Medical Research & Ethics Committee (NMRR-16-698-30042) [27]. For the present study, approval for the use of NHMS 2017 data was obtained from the Ministry of Health Malaysia. Ethical approvals for this study were obtained from both the Universiti Kebangsaan Malaysia Research Ethics Committee (FF-2021-042) and the Ministry of Health Malaysia Medical Research & Ethics Committee (NMRR-20-2743-57493). This study utilized secondary data without personal identifier information. The data are anonymous and thus the need for consent has been waived by the research ethics committee.

## Results

A total of 22,228 respondents aged 13–18 years with the mean age of 14.99 years (SD = 1.42) were included in the study. Table 1 shows the respondents’ sociodemographic characteristics. Approximately half of the respondents were female (52.7%), slightly over half of the respondents were schooling in urban areas (57.2%) and majority of the respondents were Malay (76.6%). Approximately one-tenth of the respondents (13.9%) were current smokers, while 7.7% and 2.7% were current alcohol users and current drug users, respectively.

**Table 1:** Sociodemographic characteristics and lifestyle risk behaviours among in-school adolescents in West Malaysia.

Factors	*n*	%
**Sociodemographic characteristics**		
**Gender**		
Male	10512	47.3
Female	11716	52.7
**Age (years) [Mean (SD): 14.99 (1.42)]**		
13–15	13559	61.0
16–18	8669	39.0
**Ethnicity**		
Malay	17035	76.6
Non-Malay	5193	23.4
**Locality**		
Urban	12711	57.2
Rural	9517	42.8
**Lifestyle risk behaviours**		
**Current smoker**		
Yes	3081	13.9
No	19141	86.1
**Current alcohol use**		
Yes	1705	7.7
No	20515	92.3
**Current drug use**		
Yes	608	2.7
No	21611	97.3

Abbreviation: SD–Standard deviation

**Fig 1:**
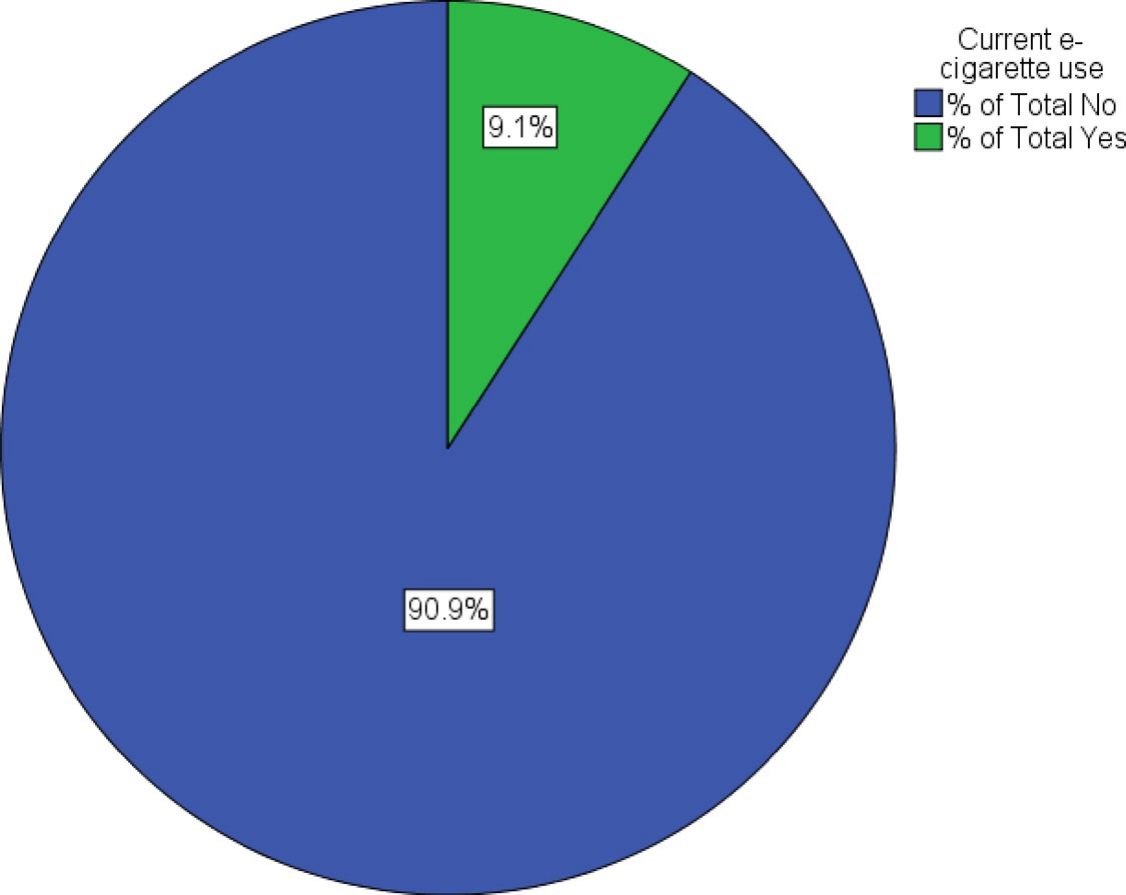
Prevalence of current e-cigarette use among in-school adolescents in West Malaysia.

The prevalence of current e-cigarette use among the respondents was 9.1% (Fig 1). Higher prevalence was observed among males (15.9%; 95% CI: 14.32, 17.56), older respondents (16–18 years) (10.1%; 95% CI: 8.69, 11.75), Malay respondents (10.0%; 95% CI: 9.03, 11.10) and respondents who were schooling in rural areas (10.4%; 95% CI: 8.96, 12.05) (Table 2). The prevalence of current e-cigarette use was higher among current smokers (45.4%; 95% CI: 42.86, 47.96), current alcohol users (28.5%; 95% CI: 23.46, 34.11), current drug users (63.9%; 95% CI: 55.78, 71.38), adolescents whose parents were not married and living together (11.6%; 95% CI: 9.96, 13.57) and adolescents whose parent(s) used any tobacco product (11.7%; 95% CI: 10.41, 13.20).

**Table 2:** Prevalence of current e-cigarette use according to sociodemographic characteristics, lifestyle risk behaviours and parental factors among in-school adolescents in West Malaysia.

Factors	*n*	Prevalence (%)	95% CI	Chi-square value	*p* value
**Overall**	1850	9.1	8.17, 10.12		
**Sociodemographic characteristics**					
**Gender**					
Male	1610	15.9	14.32, 17.56	340.63	<0.001
Female	240	2.5	1.99, 3.05		
**Age (years)**					
13–15	1033	8.4	7.33, 9.66	3.59	0.060
16–18	817	10.1	8.69, 11.75		
**Ethnicity**					
Malay	1561	10.0	9.03, 11.10	9.69	0.002
Non-Malay	289	6.7	5.17, 8.66		
**Locality**					
Urban	949	8.2	7.02, 9.58	4.40	0.037
Rural	901	10.4	8.96, 12.05		
**Lifestyle risk behaviours**					
**Current smoker**					
Yes	1368	45.4	42.86, 47.96	2798.20	<0.001
No	482	2.7	2.36, 3.19		
**Current alcohol use**					
Yes	468	28.5	23.46, 34.11	181.43	<0.001
No	1382	7.2	6.44, 8.09		
**Current drug use**					
Yes	396	63.9	55.78, 71.38	583.72	<0.001
No	1454	7.2	6.45, 8.03		
**Parental factors**					
**Parental marital status**					
Married & living together	1383	8.5	7.66, 9.51	20.09	<0.001
Others	460	11.6	9.96, 13.57		
**Parental tobacco use**					
None	665	6.3	5.48, 7.31	80.04	<0.001
One or both parents	977	11.7	10.41, 13.20		
**Had parental supervision in the past 30 days**					
Yes	268	10.2	8.55, 12.01	2.13	0.147
No	1579	8.9	7.98, 10.00		

Abbreviations: CI–Confidence interval

S1 Table shows the simple logistic regression analysis results of the factors associated with current e-cigarette use among the respondents. Multiple logistic regression analysis (Table 3) showed that male respondents had higher odds (AOR 4.74; 95% CI: 4.02, 5.60) for current e-cigarette use. The odds of current e-cigarette use were also higher among older respondents (AOR 1.41; 95% CI: 1.24, 1.60), Malays (AOR 2.68; 95% CI: 2.17, 3.32) and respondents who were schooling in urban areas (AOR 1.35; 95% CI: 1.19, 1.54). Additionally, current smokers (AOR 15.04; 95% CI: 13.19, 17.15), current alcohol users (AOR 2.68; 95% CI: 2.09, 3.44), current drug users (AOR 5.34; 95% CI: 3.99, 7.16), those whose parents were not married and living together (AOR 1.22; 95% CI: 1.04, 1.43) and those whose parent(s) used any tobacco product (AOR 1.49; 95% CI: 1.31, 1.69) were more likely to be current e-cigarette users.

**Table 3:** Factors associated with current e-cigarette use among in-school adolescents in West Malaysia.

Factors	Multiple logistic regression^a^	
	Adjusted OR (95% CI)	*p* value
**Sociodemographic characteristics**		
**Gender**		
Female	1	
Male	4.74 (4.02, 5.60)	**<0.001** **
**Age (years)**		
13–15	1	
16–18	1.41 (1.24, 1.60)	**<0.001** **
**Ethnicity**		
Non-Malay	1	
Malay	2.68 (2.17, 3.32)	**<0.001** **
**Locality**		
Rural	1	
Urban	1.35 (1.19, 1.54)	**<0.001** **
**Lifestyle risk behaviours**		
**Current smoker**		
No	1	
Yes	15.04 (13.19, 17.15)	**<0.001** **
**Current alcohol use**		
No	1	
Yes	2.68 (2.09, 3.44)	**<0.001** **
**Current drug use**		
No	1	
Yes	5.34 (3.99, 7.16)	**<0.001** **
**Parental factors**		
**Parental marital status**		
Married & living together	1	
Others	1.22 (1.04, 1.43)	0.013*
**Parental tobacco use**		
None	1	
One or both parents	1.49 (1.31, 1.69)	**<0.001** **

*p<0.05

**p<0.001

^a^Forward multiple logistic regression was applied. 19 two-way interactions were detected.

Multicolinearity was checked (VIF<10). Holsmer-Lemmeshow test *p* = 0.238, classification table (overall correctly classified percentage = 93.3%) and receiver operating characteristic (ROC) curve (area under ROC curve = 90.9%).

**Fig 2:**
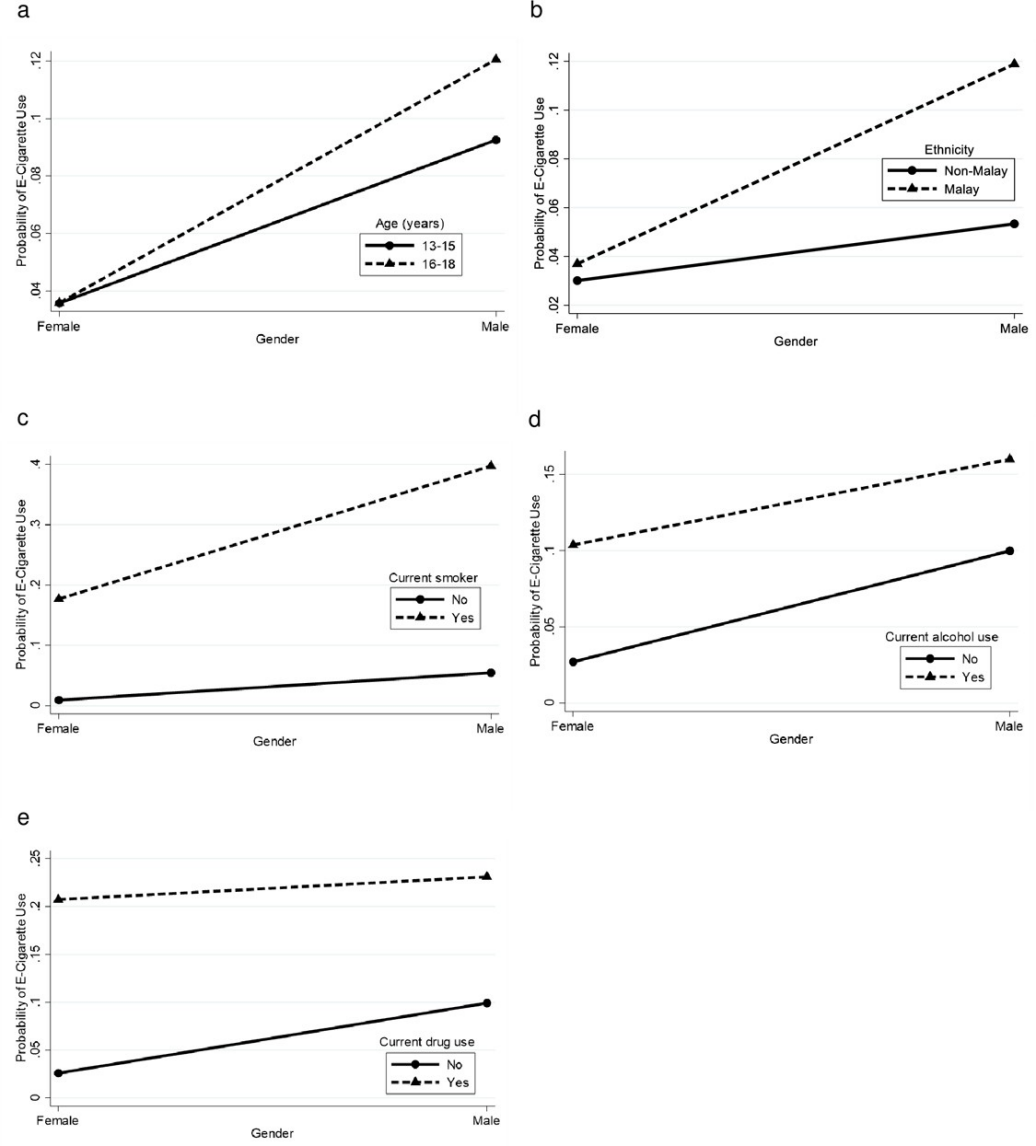
Probability of current e-cigarette use. (a) Gender-age: male (13–15 = 9.3%, 16–18 = 12.1%), female (13–15 = 3.6%, 16–18 = 3.6%). (b) Gender-ethnicity: male (non-Malay = 5.3%, Malay = 11.9%), female (non-Malay = 3.0%, Malay = 3.7%). (c) Gender-current smoker: male (no = 5.5%, yes = 39.7%), female (no = 0.9%, yes = 17.7%). (d) Gender-current alcohol use: male (no = 10.0%, yes = 16.0%), female (no = 2.7%, yes = 10.4%). (e) Gender-current drug use: male (no = 9.9%, yes = 23.1%), female (no = 2.6%, yes = 20.7%).

**Fig 3:**
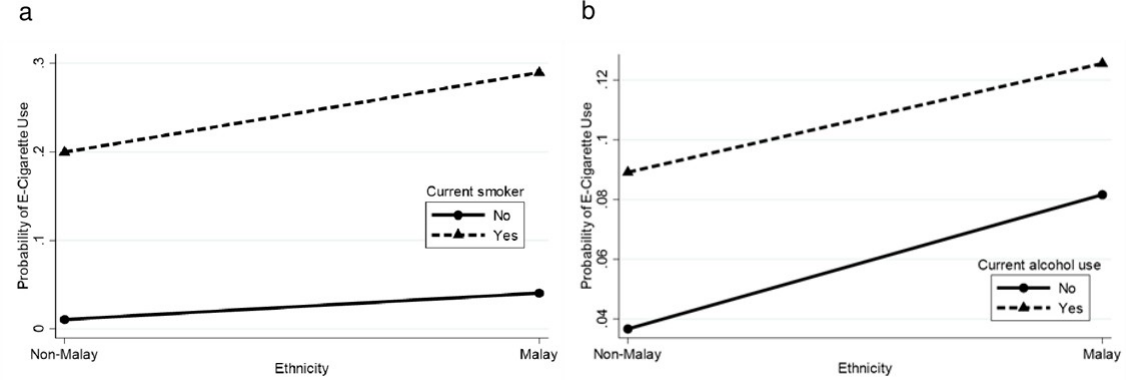
Probability of current e-cigarette use. (a) Ethnicity-current smoker: non-Malay (no = 1.1%, yes = 20.0%), Malay (no = 4.0%, yes = 29.0%). (b) Ethnicity-alcohol use: non-Malay (no = 3.7%, yes = 8.9%), Malay (no = 8.2%, yes = 12.6%).

**Fig 4:**
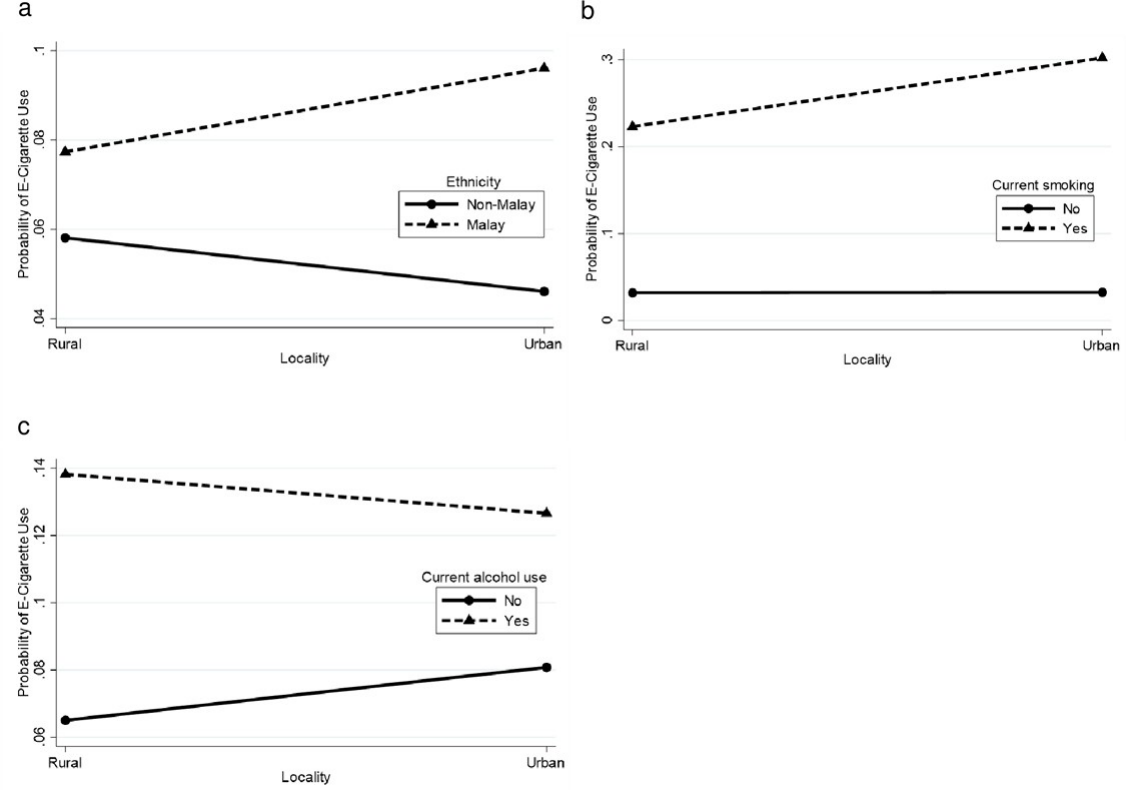
Probability of current e-cigarette use. (a) Locality-ethnicity: rural (non-Malay = 5.8%, Malay = 7.7%), urban (non-Malay = 4.6%, Malay = 9.6%). (b) Locality-smoker: rural (no = 3.2%, yes = 22.3%), urban (no = 3.3%, yes = 30.2%). (c) Locality-current alcohol use: rural (no = 6.5%, yes = 13.8%), urban (no = 8.1%, yes = 12.7%).

**Fig 5:**
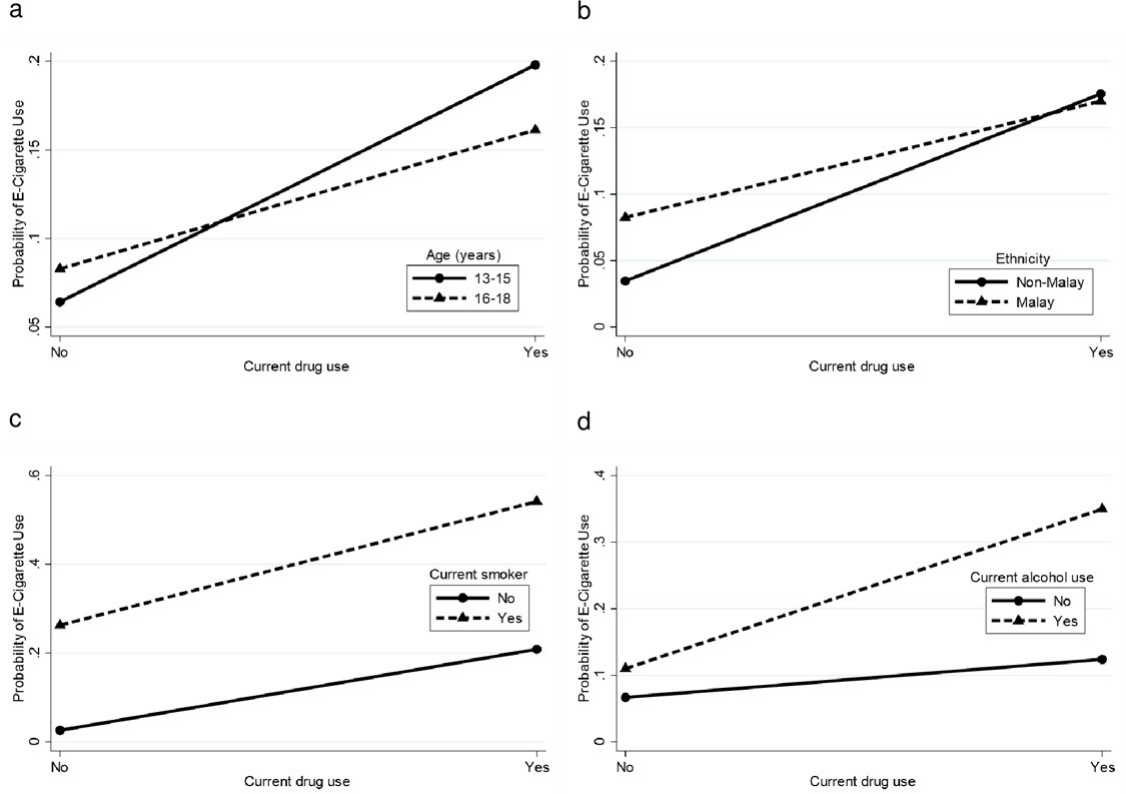
Probability of current e-cigarette use. (a) Current drug use-age: no (13–15 = 6.4%, 16–18 = 8.3%), yes (13–15 = 19.8%, 16–18 = 16.1%). (b) Current drug use-ethnicity: no (non-Malay = 3.5%, Malay = 8.2%), yes (non-Malay = 17.6%, Malay = 17.0%). (c) Current drug use-current smoker: no (yes = 26.3%, no = 2.6%), yes (yes = 54.2%, no = 20.9%). (d) Current drug use-current alcohol use: no (yes = 11.0%, no = 6.7%), yes (yes = 35.0%, no = 12.4%).

**Fig 6:**
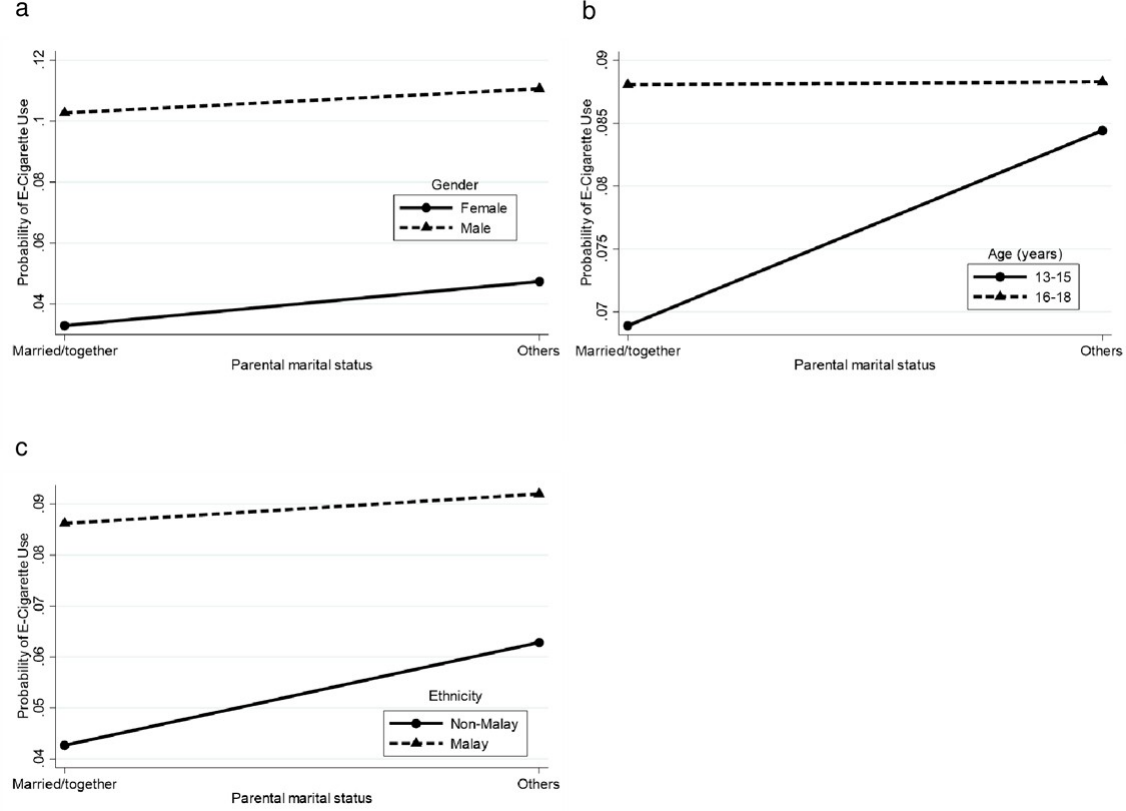
Probability of current e-cigarette use. (a) Parental marital status-gender: married and living together (male = 10.3%, female = 3.3%), others (male = 11.1%, female = 4.7%). (b) Parental marital status-age: married and living together (13–15 = 6.9%, 16–18 = 8.8%), others (13–15 = 8.4%, 16–18 = 8.8%). (c) Parental marital status-ethnicity: married and living together (non-Malay = 4.3%, Malay = 8.6%), others (non-Malay = 6.3%, Malay = 9.2%).

**Fig 7:**
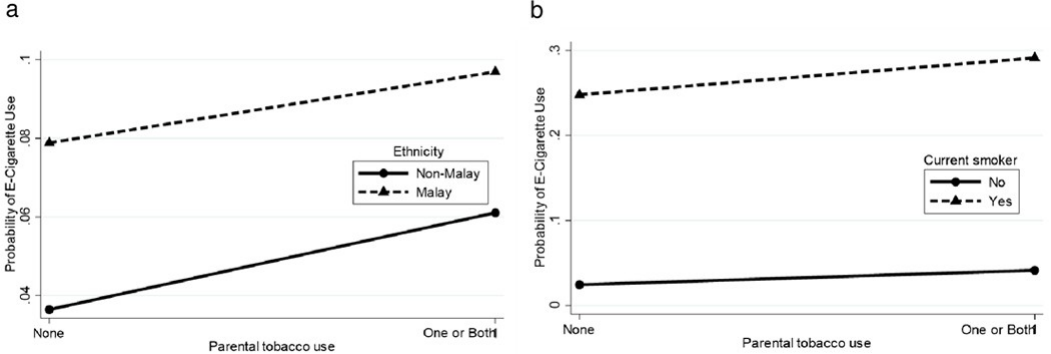
Probability of current e-cigarette use. (a) Parental tobacco use-ethnicity: one or both parents (non-Malay = 6.1%, Malay = 9.7%), none (non-Malay = 3.6%, Malay = 7.9%). (b) Parental tobacco use-current smoker: one or both parents (yes = 29.1%, no = 4.1%), none (yes = 24.8%, no = 2.4%).

There were significant interactions between: i) gender with age, ethnicity, current smoking, current alcohol use and current drug use, ii) ethnicity with current smoking and current alcohol use, iii) locality with ethnicity, current smoking and current alcohol use, iv) current drug use with age, ethnicity, current smoking and current alcohol use, v) parental marital status with gender, age and ethnicity, and vi) parental tobacco use with ethnicity and current smoking. S2 Table depicts the probability for current e-cigarette use by interactions between various combinations of sociodemographic characteristics, lifestyle risk behaviours and parental factors. These probabilities were plotted into graph (Figs 2–7).

The gender-age interaction (Fig 2A) shows that older adolescents had a greater probability of current e-cigarette use. Age differences for current e-cigarette use had a greater impact in the high-risk group [male (12.1% - 9.3% = 2.8% difference)] and lower effect in the low-risk group [female (3.6% - 3.6% = 0.0%)]. The gender-ethnicity interaction (Fig 2B) shows that Malay adolescents had a greater probability of current e-cigarette use. Ethnicity differences had greater effect for current e-cigarette use in the high-risk group [male (6.6% difference)] and lower effect in the low-risk group [female (0.7% difference)].

The gender-current smoker interaction (Fig 2C) shows that current smokers had a greater probability of current e-cigarette use. Smoking status differences had a greater effect for current e-cigarette use in the high-risk group [male (34.2% difference)] and a lower effect in the low-risk group [female (16.8% difference)]. Meanwhile, Fig 2D shows that current alcohol users had a higher probability of current e-cigarette use. The effect of alcohol use differences for current e-cigarette use was greater in the low-risk group [female (7.7% difference)] than the high-risk group [male (6.0% difference)]. Fig 2E also shows that current drug users had a greater probability of current e-cigarette use. The effect of drug use differences for current e-cigarette use was greater in the low-risk group [female (18.1% difference)] than the high-risk group [male (13.2% difference)].

In the ethnicity-current smoker interaction (Fig 3A), current smokers had a higher probability of current e-cigarette use. The interaction also shows that smoking status differences for current e-cigarette use had a greater effect in the high-risk group [Malay (25.0% difference)] than the low-risk group [non-Malay (18.9% difference)]. The ethnicity-current alcohol use interaction (Fig 3B) shows that current alcohol users had a higher probability of current e-cigarette use. The alcohol use differences had a greater effect for current e-cigarette use in the low-risk group [non-Malay (5.2% difference)] than the high-risk group [Malay (4.4% difference)].

For the locality–ethnicity interaction (Fig 4A), Malay respondents had a higher probability for current e-cigarette use. Ethnicity differences had a greater effect for current e-cigarette use in the high-risk group [urban (5.0% difference)] and a smaller effect in the low-risk group [rural (1.9% difference)]. For the locality–current smoking interaction (Fig 4B), current smokers had a higher probability of current e-cigarette use. Smoking status differences had a greater effect in the high-risk group [urban (26.9% difference)] and a smaller effect in the low-risk group [rural (19.1% difference)]. For the locality–current alcohol use interaction (Fig 4C), the probability of current e-cigarette use was higher for current alcohol users. The alcohol use differences had a greater effect in the low-risk group [rural (7.3% difference)] and a smaller effect in the high-risk group [urban (4.6% difference)].

For the current drug use-age interaction (Fig 5A), there is a cross over interaction where younger adolescents had higher probability of current e-cigarette use in the high-risk group (current drug users) but lower probability in the low-risk group (non-current drug users). This is in contrast to the probability of current e-cigarette use among older adolescents. The current drug use-ethnicity interaction (Fig 5B) shows that the ethnicity differences had a greater effect for current e-cigarette use in the low-risk group [non-current drug users (4.7% difference)] and a smaller effect in the high-risk group [current drug users (0.6% difference)]. The current drug use-current smoker interaction (Fig 5C) shows that the probability of current e-cigarette use was higher among current smokers. Smoking status differences had a greater effect for current e-cigarette use in the high-risk group [current drug users (33.3% difference)] than the low-risk group [non-current drug users (23.7% difference)]. The current drug use-current alcohol use (Fig 5D) interaction shows that current alcohol users had a higher probability of current e-cigarette use. Alcohol use difference had a greater effect for current e-cigarette use in the high-risk group [current drug users (22.6% difference)] than the low-risk group [non-current drug users (4.3% difference)].

For the parental marital status-gender interaction (Fig 6A), male adolescents had a higher probability of current e-cigarette use. Gender differences had almost similar effects in both the high-risk group [others (6.4% difference)] and low-risk group [married and living together (7.0% difference)], indicating that the impact of gender was independent of parental marital status. The parental marital status-age interaction (Fig 6B) shows that older adolescents had higher probability of current e-cigarette use. The age difference had a greater effect for current e-cigarette use in the low-risk group [married and living together (1.9% difference)] and a smaller effect in the high-risk group [others (0.4% difference)]. The parental marital status-ethnicity interaction (Fig 6C) shows that Malay adolescents had higher probability of current e-cigarette use. Ethnicity differences had a greater effect for current e-cigarette use in the low-risk group [married and living together (4.3% difference)] and a smaller effect in the high-risk group [others (2.9% difference)].

For the parental tobacco use-ethnicity interaction (Fig 7A), Malay adolescents had a higher probability of current e-cigarette use. Ethnicity difference had almost similar effects for current e-cigarette use in the high-risk group [one or both parents (3.6% difference)] and low-risk group [none (4.3% difference)], indicating that the impact of ethnicity was independent of parental tobacco use. The parental tobacco use-current smoker interaction (Fig 7B) shows that current smokers had a higher probability of current e-cigarette use. Smoking status differences had a greater effect for current e-cigarette use in the high-risk group [one or both parents (25.0% difference)] than the low-risk group [none (22.4% difference)].

## Discussion

The present study reports the most recent prevalence of current e-cigarette use among in-school adolescents in West Malaysia. We found that almost one-tenth of in-school adolescents in West Malaysia are current e-cigarette users. The prevalence is lower than that reported among adolescents in Canada (11.0%) [33] and Indonesia (11.8%) [13], but is higher than that among adolescents in Thailand (6.7%) [34] and Laos (4.3%) [35]. The differences in prevalence could be due to the effect of different e-cigarette regulations implemented in these countries. For example, Thailand has banned the importation, sale and services of e-cigarettes [36]. On the other hand, in Malaysia, only e-cigarettes that contain nicotine are regulated under the Poison Act 1952, while e-cigarettes without nicotine can be sold without restriction [36, 37].

Nevertheless, the prevalence of current e-cigarette use among in-school adolescents in Malaysia in the present study is higher compared to a report in 2012 (1.2%) [23], but similar as reported in 2016 (9.1%) [24]. In contrast to increasing trend observed in other countries [3, 38], the similar prevalence of present study and the latter report could be due to the implementation of ban on e-cigarette use in all government schools in Malaysia. The ban was stated in a circular issued by the Ministry of Education in 2015 [39].

We found that male respondents, older adolescents, Malays, respondents who were schooling in urban areas, current smokers, current alcohol users, current drug users, respondents whose parents were not married and living together and respondents whose parent(s) used any tobacco product were more likely to be current e-cigarette users. All these nine factors have interactions with other factors. The nineteen interactions between factors in this study are discussed as the probability of current e-cigarette use.

The interaction of gender with age shows a greater probability of current e-cigarette use among older adolescents. Studies conducted in the United States [11] and Malaysia [6] consistently reported that older adolescents were more likely to use e-cigarettes. As peer relationship among adolescents become more important during mid-adolescence, they might start using e-cigarettes during this period with the aim to achieve social acceptance [40]. The lower impact of age differences for current e-cigarette use in the low-risk group (female) is in keeping with the pattern of current e-cigarette use among Malaysian adolescents as reported in a nationwide survey [24]. The report indicated that the prevalence of current e-cigarette use among female adolescent was similar between the younger (2.1%) and older age group (2.6%), while the prevalence of current e-cigarette use among male adolescents differ markedly between age groups (younger: 7.5%; older: 26.5%) [24].

The gender-ethnicity interaction shows a greater probability of current e-cigarette use among Malay adolescents, which is in line with that of another recent survey among Malaysian adolescents [6]. The lower effect of ethnicity differences for current e-cigarette use in the low-risk group (female) can be partly due to that fact that smoking among females is not a norm in Malaysia and e-cigarette use is similar to smoking [41]. A nationwide survey among Malaysian adults reported that the prevalence of smoking was low in females regardless of ethnicity [42]. Malay male adults reported higher prevalence of smoking (42.8%), compared to Chinese (27.4%) and Indian (32.3%) [42]. Similar pattern might be observed in adolescent e-cigarette use as they tend to imitate their parents’ smoking behaviours [43].

The interaction of gender and current smoking shows a greater probability of current e-cigarette use among current smokers. This finding is consistent with that of other studies in Indonesia [13] and Malaysia [6]. In view of high prevalence of dual use of e-cigarettes and conventional cigarettes, it could be possible that adolescents were using e-cigarettes to help them quit smoking [44]. The different effect of smoking status differences for current e-cigarette use between males and females is in agreement with a study which reported that gender moderates the association between e-cigarette use and smoking [45]. Females who were non-smokers were significantly less likely to use e-cigarettes than males who were non-smokers [45].

The gender-current alcohol use interaction and gender-current drug use interaction show that current alcohol users and current drug users had higher probability of current e-cigarette use. These findings are explainable as local [46] and overseas [47] studies have proven that alcohol, tobacco and drug use tend to cluster among adolescents. Additionally, there is particular concern that e-cigarettes can be used to deliver cannabinoids and other illicit drugs [3]. A study conducted among adolescents in the United States found that almost one in five of e-cigarette users were vaporizing cannabis using e-cigarettes [48]. The effects of both alcohol use differences and drug use differences for current e-cigarette use were greater in the low-risk group (female). Our findings are in line with a study that determined that gender moderates the relationship between e-cigarette use and other risk behaviours, such as smokeless tobacco use, alcohol use and drug use [18].

In the ethnicity-current smoker interaction, current smokers had a higher probability of current e-cigarette use, while the effect of smoking status differences for current e-cigarette use was greater among Malay adolescents. In line with our findings, a study reported that both ethnicity (Malay) and smoking are associated with increased odds of using e-cigarettes among adolescents in Malaysia. The greater effect in the high-risk group is explainable as Malay adolescents had higher odds of current e-cigarette use compared to non-Malay adolescents (Indian, Chinese and others) [6].

The interaction of ethnicity and current alcohol use shows a higher probability of current e-cigarette use among current alcohol users. Apart from the fact that alcohol, tobacco and drug use tend to cluster among adolescents [46, 47], study had highlighted that adolescents who were e-cigarette users were more likely to be alcohol drinkers, which could be due to their tendency to exhibit risky behaviours and engage in mood-enhancing behaviours [12]. The greater effect of alcohol use differences for current e-cigarette use among non-Malay is possibly due to the higher prevalence of alcohol drinking among non-Malays, including Chinese, Indian and the indigenous people of Malaysia [49]. In addition, the indigenous people of Malaysia are more likely to practice hazardous or risky drinking [49], while these problematic alcohol users have a higher likelihood of using e-cigarettes owing to the dopaminergic reward system that is activated with the use of both alcohol and nicotine [50].

For the locality–ethnicity interaction, Malay respondents had a higher probability for current e-cigarette use. Ethnicity differences had a greater effect for current e-cigarette use in the high-risk group (urban), which could be partly explained by easier access to internet, as websites serve as platform for information and sales of e-cigarettes [51]. For the locality–current smoking interaction, current smokers had a higher probability of current e-cigarette use. Smoking status differences had a greater effect for current e-cigarette use in the high-risk group (urban), which is in line with a recent study which reported that urban current smokers were more likely than rural current smokers to be e-cigarette users. The possible reasons for the greater risk of e-cigarette use among urban current smokers are the differences in advertising, accessibility or socioeconomics [19].

For the locality–current alcohol use interaction, the probability of current e-cigarette use was higher for current alcohol users, while the effect of alcohol use differences for current e-cigarette use was greater in the low-risk group (rural). This finding is explainable as rural dwellers in Malaysia are more likely to be heavy alcohol drinkers [52], while study had reported that problematic alcohol users are more likely to use e-cigarettes due to the activation of the dopaminergic reward system when using both alcohol and nicotine [50]. For the current drug use-age interaction, younger adolescents who were current drug users had higher probability of current e-cigarette use, while those who were non-current drug users had lower probability of current e-cigarette use. Similar to our findings, a recent study conducted among adolescents in the United States found that younger e-cigarette users were more likely to use drugs compared with older adolescents [18]. Thus, age is an important aspect when screening for e-cigarette use and substance use in adolescents.

The interaction of drug use and ethnicity shows a greater effect for current e-cigarette use among non-current drug users. In contrast to our findings, local studies have reported that the use of substances was less likely to cluster in Malay compared to non-Malay adolescents [46, 53]. With the smaller effect of ethnicity differences among current drug users, further study is needed to explore the nature of drug influence in Malay and non-Malay adolescents. The interaction between current drug use with current smoking and current alcohol use show a higher probability of current e-cigarette use among current smokers and current alcohol users. The greater effects of smoking status differences and alcohol use differences for current e-cigarette use among current drug users might be expected, as local [46] and overseas studies [47] have proven that alcohol, tobacco and drug use tend to cluster among adolescents.

For the parental marital status-gender interaction, male adolescents had a higher probability of current e-cigarette use. Studies conducted in Hong Kong [10], Korea [12] and Malaysia [6] have consistently found that male adolescents have higher odds of e-cigarette use. Our findings can be explained by the fact that male adolescents are more likely than female adolescents to think that using e-cigarettes are safer than smoking cigarettes [54]. Additionally, male adolescents also experience higher exposure to e-cigarette advertisements online, which are accompanied by the unproven health benefits of e-cigarettes [55]. The similar effects of gender differences for current e-cigarette use in the high-risk group (others) and low-risk group (married and living together) could be due to the fact that both parental marital status [16] and gender [6] are associated with e-cigarette use among adolescents.

The interaction of parental marital status and age shows a higher probability of current e-cigarette use among older adolescent. The greater effect of age differences for current e-cigarette use among the low-risk group (married and living together) is mainly due to the markedly lower probability of current e-cigarette use in the low-risk group than in the high-risk group among younger adolescents. In line with our finding, a study conducted in the United States reported that adolescents who experience family instability are at higher risk of developing poor-quality peer networks, which contribute to problem behaviour. The association between family instability and problem behaviour was also found to be stronger for younger adolescents [56].

The parental marital status-ethnicity interaction shows that Malay adolescents had higher probability of current e-cigarette use. The reason of such finding could be due to the highest prevalence of e-cigarette use among adults of Malay ethnicity compared to other ethnicities [57], as adolescents tend to imitate their parents’ smoking behaviour [43]. The greater effect of ethnicity differences for current e-cigarette use among the low-risk group (married and living together) could be due to the reason that majority of ever-married adults remain married in Malaysia (male: 96.3%, female: 90.1%) and a smaller proportion of them are divorced/ separated or widowed [58].

For the parental tobacco use-ethnicity interaction, those whose parent/s used any tobacco product had higher probability of current e-cigarette use. Our findings substantiate the findings of studies that reported a significant association between parental tobacco product use and current e-cigarette use among adolescents [14, 59]. This finding can be explained by the fact that adolescents tend to imitate their parents’ smoking behaviours [43]. In addition, smoking parents might face difficulties to stop their children from using e-cigarettes due to the inconsistency between their message and behaviour. The parental tobacco use-current smoker interaction shows a greater effect of smoking status differences for current e-cigarette use among the high-risk group (one or both parents). This finding might be expected as studies has clearly shown that both adolescent smoking [6, 13] and parental tobacco use [14, 59] are associated with e-cigarette use among adolescents.

There are limitations to our study. First, temporal causality cannot be determined, as our study utilized cross-sectional data. Second, data were self-reported, and there is the possibility of imprecise reporting due to recall bias. Despite certain methodological limitations, our study used a large representative sample of adolescents from West Malaysia, and therefore produces more generalisable findings.

## Conclusion

The present study found that almost one-tenth of Malaysian adolescents were current e-cigarette users. Current e-cigarette use is associated with sociodemographic characteristics, lifestyle risk behaviours and parental factors. Our findings provide new insight into the interactions between various combinations of sociodemographic characteristics (gender, age, ethnicity, locality), lifestyle risk behaviours (current smoking, current alcohol use, current drug use) and parental factors (parental marital status, parental tobacco use) that affect the probability of current e-cigarette use among West Malaysian adolescents. Efforts to tackle e-cigarette use should target sociodemographic characteristics, lifestyle risk behaviours and parental factors such as smoking cessation intervention for parents. Adolescents who are current e-cigarette users were more likely to engage in other lifestyle risk behaviours, including smoking, alcohol and drug use. Thus, there is a need to monitor these risk behaviours simultaneously among adolescents. The need for smoking cessation intervention must be emphasized. These efforts should be complemented with findings from the sociodemographic characteristic, lifestyle risk behaviour and parental factor interactions. Policies, programmes and interventions must be strengthened and tailored towards the needs of adolescents who are at risk of e-cigarette use.

## Supporting information

S1 Table Preliminary factors associated with current e-cigarette use among in-school adolescents in West Malaysia. (DOCX)

S2 Table Probability for current e-cigarette use according to interactions between sociodemographic characteristics, lifestyle risk behaviours and parental factors. (DOCX)
