# Supplementary material for: Current e-cigarette use among in-school adolescents in West Malaysia: Examining the interactions between sociodemographic characteristics and lifestyle risk behaviours
Source: PLoS One. 2022 Jan 31;17(1):e0263355. doi: 10.1371/journal.pone.0263355 (PMC8803165; doi:10.1371/journal.pone.0263355)
Supplement: S1 Table — (DOCX) [file pone.0263355.s001.docx]

**S1 Table. Preliminary factors associated with current e-cigarette use among in-school adolescents in West Malaysia**

| **Factors** | **Simple logistic regression** | |
| --- | --- | --- |
|  | **Crude OR**  **(95% CI)** | ***p* value** |
| **Sociodemographic characteristics** |  |  |
| **Gender**  Female  Male | 1  8.65 (7.53, 9.94) | **<0.001**** |
| **Age (years)**  13-15  16-18 | 1  1.26 (1.15, 1.39) | **<0.001**** |
| **Ethnicity**  Non-Malay  Malay | 1  1.71 (1.50, 1.95) | **<0.001**** |
| **Locality**  Rural  Urban | 1  0.77 (0.70, 0.85) | **<0.001**** |
| **Lifestyle risk behaviours** |  |  |
| **Current smoker**  No  Yes | 1  31.00 (27.63, 34.78) | **<0.001**** |
| **Current alcohol use**  No  Yes | 1  5.26 (4.67, 5.93) | **<0.001**** |
| **Current drug use**  No  Yes | 1  26.26 (22.02, 31.31) | **<0.001**** |
| **Parental factors** |  |  |
| **Parental marital status**  Married & living together  Others | 1  1.69 (1.51, 1.89) | **<0.001**** |
| **Parental tobacco use**  None  One or both parents | 1  2.03 (1.83, 2.25) | **<0.001**** |
| **Had parental supervision in the past 30 days**  No  Yes | 1  1.12 (0.98, 1.29) | 0.092 |
