# Supplementary material for: Current e-cigarette use among in-school adolescents in West Malaysia: Examining the interactions between sociodemographic characteristics and lifestyle risk behaviours
Source: PLoS One. 2022 Jan 31;17(1):e0263355. doi: 10.1371/journal.pone.0263355 (PMC8803165; doi:10.1371/journal.pone.0263355)
Supplement: S2 Table — (DOCX) [file pone.0263355.s002.docx]

**S2 Table. Probability for current e-cigarette use according to interactions between sociodemographic characteristics, lifestyle risk behaviours and parental factors**

| **Sociodemographic characteristic or lifestyle risk behaviour or parental factor** | **Sociodemographic characteristic or lifestyle risk behaviour** | **Probability for current e-cigarette use** | |
| --- | --- | --- | --- |
|  |  | ***p* value** | **% (95% CI)** |
| **Gender**  Male  Male  Female  Female | **Age (years)**  13-15  16-18  13-15  16-18 | **<0.001**  **<0.001**  **<0.001**  **<0.001** | 9.3 (8.68, 9.83)  12.1 (11.26, 12.85)  3.6 (3.07, 4.07)  3.6 (2.94, 4.22) |
| **Gender**  Male  Male  Female  Female | **Ethnicity**  Malay  Non-Malay  Malay  Non-Malay | **<0.001**  **<0.001**  **<0.001**  **<0.001** | 11.9 (11.28, 12.49)  5.3 (4.54, 6.13)  3.7 (3.22, 4.17)  3.0 (2.34, 3.68) |
| **Gender**  Male  Male  Female  Female | **Current smoker**  Yes  No  Yes  No | **<0.001**  **<0.001**  **<0.001**  **<0.001** | 39.7 (37.60, 41.87)  5.5 (4.94, 5.99)  17.7 (14.72, 20.67)  0.9 (0.73, 1.13) |
| **Gender**  Male  Male  Female  Female | **Current alcohol use**  Yes  No  Yes  No | **<0.001**  **<0.001**  **<0.001**  **<0.001** | 16.0 (13.52, 18.46)  10.0 (9.47, 10.50)  10.4 (7.99, 12.75)  2.7 (2.29, 3.12) |
| **Gender**  Male  Male  Female  Female | **Current drug use**  Yes  No  Yes  No | **<0.001**  **<0.001**  **<0.001**  **<0.001** | 23.1 (18.99, 27.21)  9.9 (9.41, 10.42)  20.7 (14.57, 26.88)  2.6 (2.20, 2.97) |
| **Ethnicity**  Malay  Malay  Non-Malay  Non-Malay | **Current smoker**  Yes  No  Yes  No | **<0.001**  **<0.001**  **<0.001**  **<0.001** | 29.0 (27.18, 30.76)  4.0 (3.66, 4.44)  20.0 (16.52, 23.41)  1.1 (0.76, 1.37) |
| **Ethnicity**  Malay  Malay  Non-Malay  Non-Malay | **Current alcohol use**  Yes  No  Yes  No | **<0.001**  **<0.001**  **<0.001**  **<0.001** | 12.6 (10.34, 14.78)  8.2 (7.80, 8.53)  8.9 (7.63, 10.20)  3.7 (2.94, 4.39) |
| **Locality**  Rural  Rural  Urban  Urban | **Ethnicity**  Malay  Non-Malay  Malay  Non-Malay | **<0.001**  **<0.001**  **<0.001**  **<0.001** | 7.7 (7.27, 8.20)  5.8 (4.64, 6.97)  9.6 (9.08, 10.13)  4.6 (3.95, 5.27) |
| **Locality**  Rural  Rural  Urban  Urban | **Current smoker**  Yes  No  Yes  No | **<0.001**  **<0.001**  **<0.001**  **<0.001** | 22.3 (20.40, 24.21)  3.2 (2.78, 3.62)  30.2 (27.96, 32.44)  3.3 (2.88, 3.63) |
| **Locality**  Rural  Rural  Urban  Urban | **Current alcohol use**  Yes  No  Yes  No | **<0.001**  **<0.001**  **<0.001**  **<0.001** | 13.8 (11.53, 16.11)  6.5 (6.06, 6.95)  12.7 (10.66, 14.65)  8.1 (7.63, 8.53) |
| **Current drug use**  Yes  Yes  No  No | **Age (years)**  13-15  16-18  13-15  16-18 | **<0.001**  **<0.001**  **<0.001**  **<0.001** | 19.8 (16.45, 23.15)  16.1 (11.83, 20.43)  6.4 (6.01, 6.83)  8.3 (7.78, 8.79) |
| **Current drug use**  Yes  Yes  No  No | **Ethnicity**  Malay  Non-Malay  Malay  Non-Malay | **<0.001**  **<0.001**  **<0.001**  **<0.001** | 17.0 (13.95, 20.06)  17.6 (13.49, 21.62)  8.2 (7.84, 8.66)  3.5 (2.88, 4.06) |
| **Current drug use**  Yes  Yes  No  No | **Current smoker**  Yes  No  Yes  No | **<0.001**  **<0.001**  **<0.001**  **<0.001** | 54.2 (47.75, 60.55)  20.9 (14.32, 27.43)  26.3 (24.60, 27.94)  2.6 (2.38, 2.88) |
| **Current drug use**  Yes  Yes  No  No | **Current alcohol use**  Yes  No  Yes  No | **<0.001**  **<0.001**  **<0.001**  **<0.001** | 35.0 (29.88, 40.03)  12.4 (9.11, 15.69)  11.0 (9.24, 12.77)  6.7 (6.37, 7.00) |
| **Parental marital status**  Married & living together  Married & living together  Others  Others | **Gender**  Male  Female  Male  Female | **<0.001**  **<0.001**  **<0.001**  **<0.001** | 10.3 (9.76, 10.79)  3.3 (2.84, 3.73)  11.1 (9.94, 12.20)  4.7 (3.78, 5.69) |
| **Parental marital status**  Married & living together  Married & living together  Others  Others | **Age (years)**  13-15  16-18  13-15  16-18 | **<0.001**  **<0.001**  **<0.001**  **<0.001** | 6.9 (6.47, 7.30)  8.8 (8.28, 9.33)  8.4 (7.54, 9.34)  8.8 (7.67, 9.99) |
| **Parental marital status***  Married & living together  Married & living together  Others  Others | **Ethnicity**  Malay  Non-Malay  Malay  Non-Malay | **<0.001**  **<0.001**  **<0.001**  **<0.001** | 8.6 (8.22, 9.02)  4.3 (3.64, 4.90)  9.2 (8.34, 10.06)  6.3 (5.05, 7.52) |
| **Parental tobacco use**  None  None  One or both parents  One or both parents | **Ethnicity**  Malay  Non-Malay  Malay  Non-Malay | **<0.001**  **<0.001**  **<0.001**  **<0.001** | 7.9 (7.39, 8.38)  3.6 (3.00, 4.29)  9.7 (9.15, 10.23)  6.1 (5.12, 7.09) |
| **Parental tobacco use**  None  None  One or both parents  One or both parents | **Current smoker**  Yes  No  Yes  No | **<0.001**  **<0.001**  **<0.001**  **<0.001** | 24.8 (22.67, 26.89)  2.4 (2.11, 2.75)  29.1 (26.95, 31.27)  4.1 (3.64, 4.60) |
